# The Relationship between Vegetarian Diet and Sports Performance: A Systematic Review

**DOI:** 10.3390/nu15214703

**Published:** 2023-11-06

**Authors:** Juan Hernández-Lougedo, José Luis Maté-Muñoz, Pablo García-Fernández, Edurne Úbeda-D’Ocasar, Juan Pablo Hervás-Pérez, Blanca Pedauyé-Rueda

**Affiliations:** 1 Faculty of Health Sciences, Camilo José Cela University, 28692 Madrid, Spain; jlougedo@ucjc.edu (J.H.-L.); eubeda@ucjc.edu (E.Ú.-D.); jphervas@ucjc.edu (J.P.H.-P.); blanca.pedauye@ucjc.edu (B.P.-R.); 2 Department of Radiology, Rehabilitation and Physiotherapy, Complutense University of Madrid, 28040 Madrid, Spain; pablga25@ucm.es

**Keywords:** vegetarian diet, vegan diet, performance, sport and exercise

## Abstract

Introduction: In recent years, the vegetarian diet has increased in popularity among athletes. The aim of this review is to ascertain the differences in variables related to performance, nutritional intake, and health in athletes according to whether they are omnivores or vegetarians. Methodology: A literature search was carried out in different databases: PubMed, Web of Science, Dialnet, and Cochrane. The keywords used were “vegetarian diet”, “vegan diet”, “exercise”, “sport”, and “performance”. After applying different inclusion criteria, six studies were included in the review. Results: No significant differences were obtained in variables related to physical performance (adherence exercise, Vo2Máx, muscle power, and sprint test) or health (body composition, psychological well-being, and social relationships), but dietary intake was significantly higher in carbohydrates and lower in proteins in vegetarian athletes (*p* < 0.05). Conclusions: It cannot be affirmed that vegetarian subjects have a higher sports performance, for which more research should be carried out.

## 1. Introduction

The population’s diet has changed over the years, which has led to an increase in the consumption of plant-based foods in Western countries [1,2,3,4].

A vegetarian is defined as a person who does not consume any type of meat, including poultry, seafood, fish, or products containing them. However, within vegetarianism, there are different groups, some less restrictive than others. From less restrictive to more restrictive, we find the ovolactovegetarians who do include dairy products and eggs in their diet, the lactovegetarians who only include milk, and the vegans who do not include any type of food of animal origin [5,6,7] (Table 1).

The reason why people choose to consume such foods varies greatly depending on their age:

- Adolescents for reasons of animal or environmental concern.
- Adults who wish to improve their health.

Historically, this type of food is related to health, culture, and religious factors [8,9]. The origin of this diet is found in ethical–religious and medical movements, since it was used as a ritual of health and purity of the body. For example, in China, a large part of the population continues to maintain a vegetarian diet and it is also in traditional Russian medicine, in India meat is not consumed because it is considered an act of violence, and in the Buddhist religion, meat consumption was not introduced until the end of the Second World War [10].

However, in Europe, vegetarianism began in the Renaissance, and from that moment, began to expand. It was in 1847 when a group of vegetarians met and formed the first vegetarian society in Europe, which was named “Vegetarian Society” [10].

The benefits of this diet, as evidenced by the American Dietetic Association, are an improvement in health and in the prevention and treatment of pathologies. It has been shown that the chances of developing cardiovascular pathologies are lower, while some cancers have shown improvements in the biochemical parameters of the organism. It should be noted that vegetarians are not only characterized by their eating style, but also by generally engaging in more physical activity and consuming less harmful products [11].

However, this diet not only has the benefits outlined above, but it also has some negative aspects. The main disadvantage is the possibility of suffering from nutritional deficiencies, especially in vitamin B12, zinc, iron, calcium, omega-3 fatty acids, and protein [7,11].

To ascertain the effects that this diet may have on sports performance, we need to compare the nutritional contributions of the foods that make up the vegetarian diet with that of the omnivorous diet.

According to The Academy of Nutrition and Dietetics, a well-planned vegetarian diet can meet the macronutrient and energy needs of an athlete [12,13]. Vegetarian diets can vary widely in terms of calorie content and fiber, just like omnivorous diets. It is important for vegetarian athletes to focus on a balanced and nutrient-rich diet that supports their performance and overall health. As for the carbohydrate requirements of sportsmen and sportswomen, mainly in endurance sports, athletes need to replenish their glycogen stores, as the success of their sports performance will depend on this. This is why carbohydrate intake recommendations are between 3 and 12 g/kg/day depending on the volume, intensity, and type of exercise effort [11]. Vegetarian diets do not meet the daily protein recommendation, but it should be kept in mind that, in the case of athletes, the requirements are higher, and these depend on the sports: 1–1.6 g/kg in endurance sports and 1.4–2 g/kg for strength athletes [11,14]. It has been shown that people who follow a vegetarian diet can cover their protein requirements of high biological value if they consume eggs and dairy products, as well as legumes and nuts. With respect to fats, the requirement for athletes is like that of non-athletes (20–35% of total daily calories), and these should be healthy fats [11].

When planning a diet for an athlete, the total energy requirement must be taken into account, along with the basal metabolism, the energy expenditure generated by the physical exercise performed, and the thermogenic effect of food, which represents between 3% and 10% of the total energy expenditure [15]. Some of the methods used to calculate it are the equations, such as Harris–Benedict or Mifflin–St-Jeor, using electronic devices such as watches or cell phones, or direct and indirect calorimetry.

Hydration is a key factor in successful performance, as it optimizes thermoregulation during exercise [16]. Correct fluid levels should be monitored before, during, and after exercise. Ideally, fluid loss should not exceed 2%, as higher values decrease cognitive function and performance. Athletes can lose between 0.3 and 2.4 liters of body fluid per hour of exercise through sweat, depending on variables, such as environment, gender, body size, and exercise duration [15].

We must also take into account the intake of micronutrients, which, according to several studies, is deficient when a vegetarian diet is followed [17]. The main mineral that research should be focused on is iron. The type of iron suitable for vegetarians is non-heme iron, and the amount that is absorbed will depend on whether it is consumed with enhancers (vitamin C and citric acid) or inhibitors. Iron is important in an athlete’s performance as it is involved in the delivery of oxygen to the muscle. According to Fuhrman and Ferreri (2010), it is not necessary to take supplements if an adequate amount of food containing this mineral is consumed, except in cases of anemia, low ferritin, or menorrhagia. Something similar happens with zinc, which is consumed in foods that contain a large amount of this micronutrient, although such foods also inhibit its absorption. Supplementation is recommended due to its importance in the functioning of the immune system function [18]. These studies have linked vegetarian diets with vitamin B12 deficiency, which is associated with cardiovascular pathologies [18]. These same authors mention the importance of an adequate supply of vitamin D in athletes, since it is directly related to the musculoskeletal system, and the best sources of vitamin D are sun exposure and/or the consumption of fortified foods.

Injured athletes should control caloric intake to lose as little muscle mass as possible during the period when there is the least amount of movement. During this period, athletes should increase protein intake by 1.2–1.5 g/kg to aid healing tissue formation while reducing muscle loss [15].

In terms of performance in strength and endurance sports, it can be stated that the vegetarian diet provides the necessary nutrients for good performance, provided that the diet is well planned. In strength sports, protein intake is especially relevant, although with the intake of plant proteins, such as legumes, seeds, nuts, and whole grains, the recommendations can be met [5]. In addition, there are now a large number of foods enriched with micro-nutrients, which can help vegetarian or vegan athletes reach the recommended intakes.

Some of the athletes, both strength and endurance, who eat a vegetarian or vegan diet are Derek Tresize and Carla Lewis, bodybuilder and velocity athlete [19], respectively, as well as in intermittent sports, such as soccer or tennis, as we find in the cases of Saul Ñiguez and Novak Djokovic.

This review aims to assess the effects of vegetarian and omnivorous diets on various aspects of athletic performance, health-related parameters, and nutritional intake.

## 2. Materials and Methods

### 2.1. Acquisition of Evidence

For this systematic review, we followed the protocol according to the standards and guidelines of the PRISMA statement for systematic reviews and meta-analyses, which aims to improve the reporting of future systematic reviews [20].

### 2.2. Eligibility Criteria

We included articles that met the following inclusion criteria: (a) publications in the last ten years (from 2013 to 2023); (b) written in English or Spanish; (c) clinical trials and randomized controlled clinical trials using a placebo or control group; (d) relationship between diet and sports performance; (e) women of working age (intervention group performing a physiotherapeutic intervention; (f) cytokine analysis. Exclusion criteria were (i) animals were used for research. (ii) clinical trials without results or not completed. (iii) literature reviews.

### 2.3. Sources of Information

The literature search was conducted between September 2022 and September 2023. The aim of the search was to find out whether athletic performance changed as a function of the diet the athlete would take. The databases used were Web Of Science (WOS), PubMed, Cochrane, and Dialnet.

### 2.4. Search Strategies

The keywords we used for the document search were “vegetarian diet”, “vegan diet”, “performance”, “sport”, and “exercise”. The search strategy was (“vegetarian diet” OR “vegan diet”) AND (performance OR sport OR exercise). At the end of the search, we had 263 articles that met the search criteria, after reading the abstracts, methods, and objectives, exclusion criteria were applied:

- Articles with a publication date prior to 2013.
- Articles that were written in a language other than English or Spanish.
- Articles in which animals were used for research.
- Articles that were literature reviews.
- Articles that did not link diet to sports performance.

### 2.5. Data Extraction Process

An exhaustive reading and evaluation of the six studies finally selected were carried out, to which the PEDro scale was applied to assess their methodological quality, evaluating the design of the study, the source of obtaining the subjects, whether the study was randomized, whether there was concealment, whether there was blinding, and what the outcome of the study was like. The PEDro scale of the synthesis results can be found in more detail in Section 3.5.

**Figure 1:**
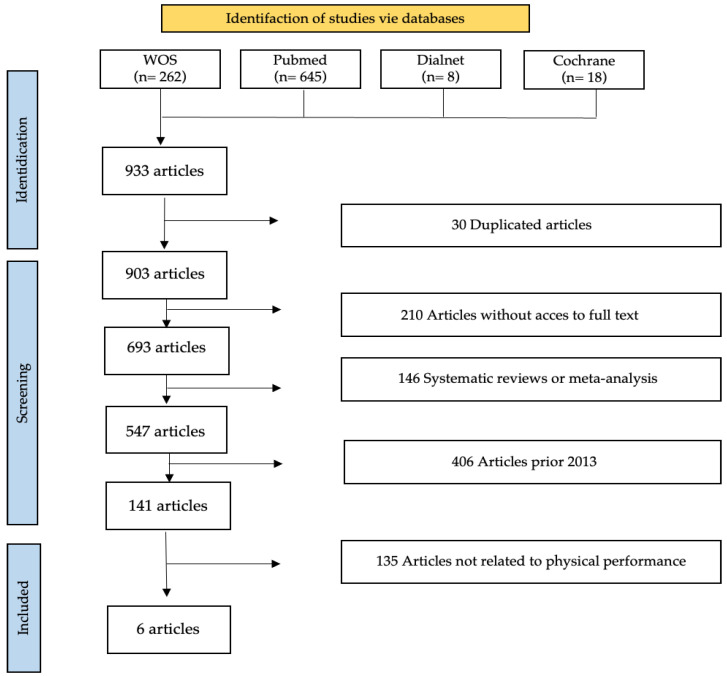
Flow diagram according to the PRISMA declaration.

In addition, the PRISMA 2020 checklist [20] was used to collect the most relevant data for each of the studies, author and year, type of study, sample characteristics, objectives, type of intervention, intervention time, diet, ergogenic aids, program, healthy variables, and performance variables. Figure 1 shows the process followed in selecting the articles used for the literature review according to PRISMA declaration. The results of the data extraction will be presented in Supplementary Materials.

### 2.6. Risk of Bias Assessment of Individual Studies

Risk of bias is a tool developed by the Cochrane Collaboration to assess the methodology of scientific evidence. It is useful in systematic reviews for the individual analysis of included CTs and RCTs. In this sense, the present systematic review has followed the Cochrane Handbook 5.1.0 [21] to assess the risk of bias.

The Cochrane Handbook 5.1.0 presents six levels of bias: selection bias, conduct bias, detection bias, attrition bias, reporting bias, and other bias. Each level has one or more specific items in a Risk of Bias table, and each item includes a description of what happened in the study and an assessment where the assignment of “low risk”, “high risk”, or “unclear risk” of bias is included [21].

### 2.7. Synthesis Methods

The synthesis methods used in the present review are the eligibility criteria that were determined in Section 2.2 of Material and Methods and the analysis of methodological quality using the PEDro scale, which is based on the Delphi checklist developed by Verhagen [22]. The checklist has a total of 11 items. The first item refers to external validity and is not considered for the final score, items 2–9 refer to internal validity, and items 10 and 11 indicate whether the statistical information provided by the authors allows for an adequate interpretation of the results.

Therefore, the maximum score is 10 points, and the minimum is 0. Only items that are answered affirmatively are scored. Studies with a score of 9–10 were of excellent methodological quality, 6–8 were of good quality, and 5 were of fair or acceptable quality. The PEDro scale can be found in more detail in Section 3.5.

Further to the synthesis measures, we assessed whether the studies included in the analysis met their objectives set at the start of the study.

### 2.8. List of Variables

Table 2 details the study variables found in the articles found for this systematic review.

## 3. Results

### 3.1. Selection of Studies

During the initial phase of the search, 933 studies were identified from different databases. After eliminating duplicates, 903 studies remained.

To refine the selection, we applied date filters (2013–2023) and chose to select only clinical trials and randomized clinical trials, which were available in English or Spanish, leaving us with 141 articles. After reviewing the titles and abstracts, 135 studies that did not fit the study topic were discarded. After a detailed reading, a total of six studies that met the inclusion criteria were selected and subjected to a qualitative analysis, using database filters for date and type of study. Mendeley was used to search for duplicates.

### 3.2. Characteristics of the Studies

Of the total articles included in this review, 33.33% were published in 2016 [23,24], 16.67% in 2018 [25], 16.67% in 2020 [26], and 33.33% in 2021 [27,28]. A total of 3363 individuals participated, with 1921 females, of whom 543 were vegetarians, 652 vegans, and 726 omnivores; and 1442 males, with 305 vegetarians, 352 vegans, and 785 omnivores.

The articles were cross-sectional studies, trial-control studies, or randomized clinical trials. Of the six studies included in this systematic review, five present a group of athletes eating a vegetarian or vegan diet and a control group composed of athletes eating an omnivorous diet. A single observational study compares subjects according to diet and sport modality, 10 km, half marathon, and marathon. More detailed information can be found in the descriptive table of each of the studies in Table 3.

### 3.3. Results of Individual Studies

The results of the data extraction will be presented in Table 3.

### 3.4. Risk of Bias in Individual Studies

A risk of bias assessment of the individual studies was performed, allowing a more accurate picture of the quality of the available evidence and the reliability of the results obtained.

The risk of bias assessment figures for each study included in this systematic review are shown below. Each figure will show the result of the risk of bias assessment for each domain assessed, allowing us to identify the strengths and weaknesses of each study. In this way, we will gain a more detailed understanding of the quality of the included studies and their impact on the overall results of the systematic review.

**Figure 2:**
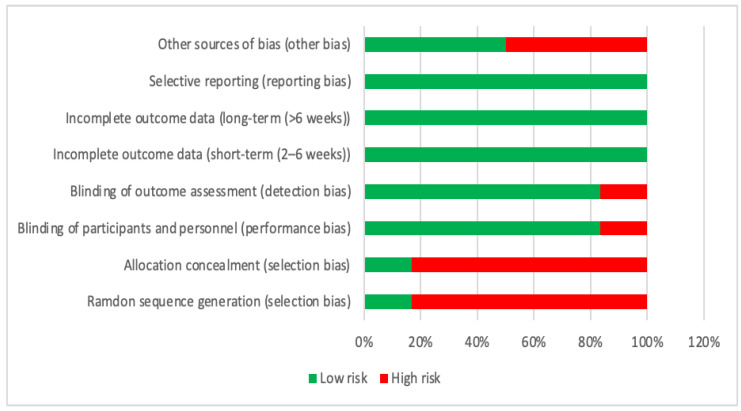
Risk of bias.

In the risk of bias graph (Figure 2), it can be seen that the incomplete short- and long-term outcome data and selective reporting is 100% low risk; while blinding of participants, personal, and outcome assessment is 83.3% low risk; other source of bias is 50% low risk; and randomized sequence generation and allocation concealment is 83.3% high risk.

In the present review, the articles with the greatest bias are those that are not randomized and that do not blind the group assignment [23,25,26,27,28]. The article with the greatest bias is due to the lack of blinding of the subjects and the evaluators, as well as other biases such as only specifying the depurative modality [25]. In some articles there are other types of biases, such as not specifying exercise variables [27] or that the calculated dietary intake was based on a 3-day recall [25,26].

**Figure 3:**
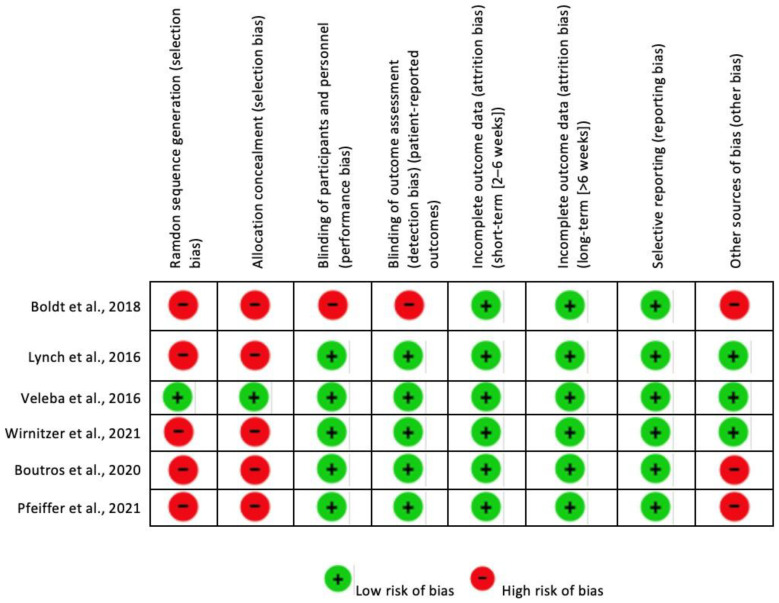
Risk of bias summary [23,24,25,26,27,28].

Furthermore, in the risk of bias summary (Figure 3), it can be seen which author and item has a low risk, unclear risk, and high risk.

### 3.5. Results of the Synthesis

The articles included in the review were assessed using the PEDro methodological quality scale, shown below in Table 4. The final score obtained ranged from 5 to 10. Five studies were classified as being of good quality and one of fair quality. The studies achieved a mean value of 6.16 ± 0.75.

## 4. Discussion

**Figure 4:**
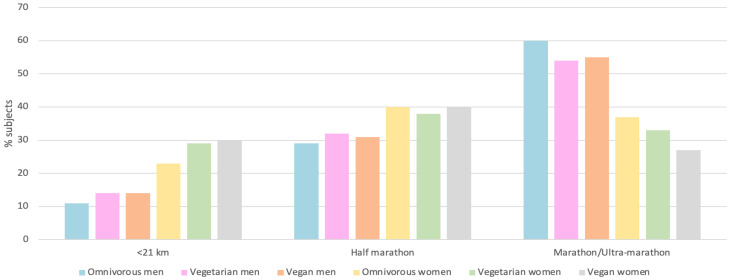
Percent of subjects performing different endurance modalities as a function of diet [27].

After analyzing the different studies, we observed that the practice of physical exercise and diet must be understood together to perform at one’s maximum. As shown in Figure 4, the percentage of subjects who performed different endurance tests was evaluated as a function of their diet, and it was observed that in the tests of less than 21 km, the vegans were the ones who performed the largest number of tests, both male and female athletes (14% and 10%, respectively); in half marathons, male vegans (32%) and female vegetarians (43%) obtained the greatest percentage; and, finally, in marathon or ultra marathon events, male and female omnivores were the ones who performed the largest number of tests (60% and 37%, respectively) [27].

### 4.1. Physical Health

**Figure 5:**
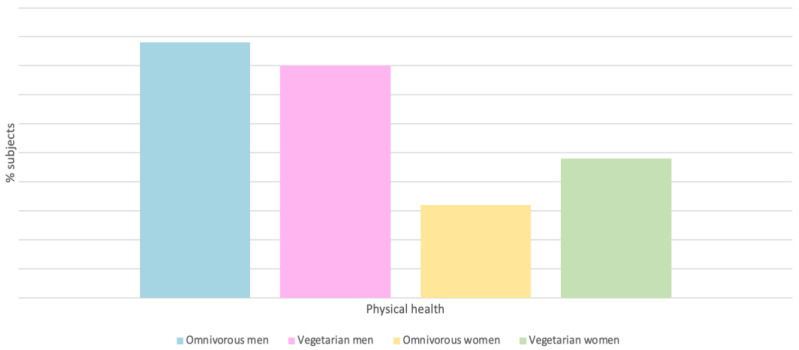
Physical health % of different subjects as a function of diet [25].

Through the quality-of-life questionnaire (WHOQOL-BREF), there is a difference in some variables related to health depending on diet and type of exercise. The results show that omnivorous women and men have better physical health, but the differences are not significant, so to say that both are adequate to maintain good physical health is not entirely accurate (Figure 5). Physical health is greater in women who run half marathons, followed by those who participate in marathons or ultra marathons, and, finally, those who run 10 km races. However, in men, physical fitness decreases gradually as the distance increases [25].

Vegetarian diets can help athletes protect themselves from degenerative and inflammatory diseases, as well as improve their body composition [29].

Other parameters related to health in the sports environment have also been studied, such as psychological well-being, the environment, and social relationships were also researched. In relation to psychological well-being and social relationships, athletes with an omnivorous diet report a higher level, although the differences are not significant. Finally, and in relation to the environment, the omnivorous subjects were also those who presented a better adaptation, with significant differences in women according to the sport modality, half marathon, and 10 km races, with the former presenting a greater adaptation to the environment [25].

### 4.2. Body Composition

**Figure 6:**
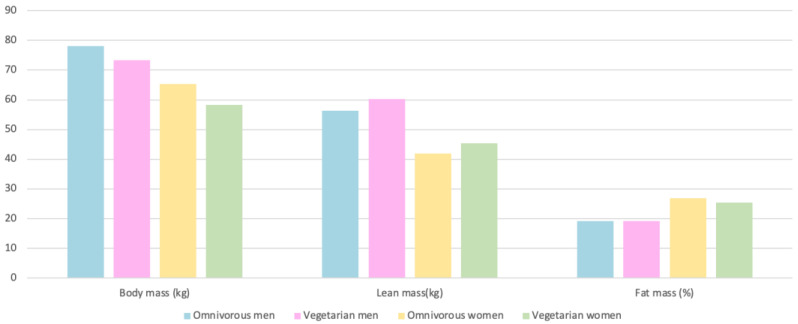
Body composition of subjects according to diet [23].

Body composition is a factor that is directly related to athletic performance, although there are no ideal values, since they vary depending on the sport (Figure 6) [23].

#### 4.2.1. Body Mass

Vegetarian athletes had body weights that were 11% higher compared to omnivores. This suggests that the body weight of vegetarians who are athletes is significantly greater than that of those who consume an omnivorous diet [23,30].

Lactovegetarians, on the other hand, had body weights that were 7.3% lower compared to omnivores. Lactovegetarians are vegetarians who consume dairy products, and they had a lower body weight compared to omnivores.

Vegetarian athletes were 11.1% more likely to fall into the “normal weight” category according to the criteria established by the World Health Organization (WHO), which defines normal weight as having a Body Mass Index (BMI) between 18.5 and 25 kg/m^2^ [31]. This suggests that vegetarian athletes were more likely to maintain a normal weight compared to omnivores.

#### 4.2.2. Lean Mass

Muscle mass was 7% lower in vegetarian athletes compared to omnivores [23]. In male endurance athletes, muscle mass was also lower in those on an ovolactovegetarian diet compared to omnivores, specifically by 1.6% [30].

#### 4.2.3. Fat Mass

In relation to fat mass in athletes, it depends on sex and diet. In men, there are no significant differences in the percentage of fat mass, while in women, omnivorous athletes had 1.4% more fat mass according to their body weight [23].

The athlete’s body composition is a factor that varies according to the competitive period that he/she is in. Both body weight and fat mass decrease as the season progresses, although skeletal muscle mass remains stable in relation to body weight. This may be due to training and competition, as well as energy intake and distribution [32].

### 4.3. Performance

On diet and exercise compliance, vegetarians had a high adherence rate of 55%, while omnivores had an adherence rate of only 32%. Sometimes, this may be because high-performance athletes are hesitant to follow these types of nutritional guidelines that would allow them to achieve the desired performance for competition [24].

#### 4.3.1. Endurance exercise

**Figure 7:**
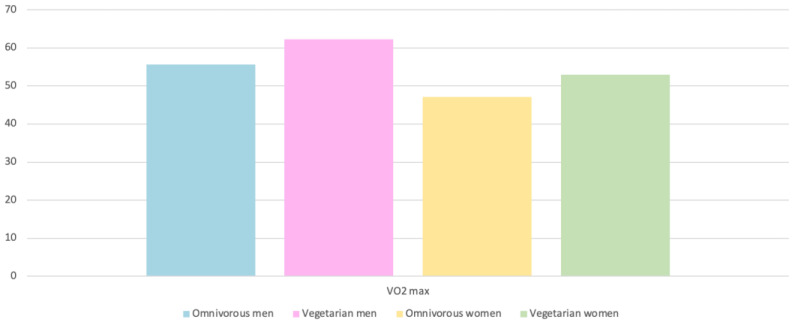
VO2 max of the subjects according to the type of diet [23].

As shown in Figure 7, vegetarian subjects performed 20% more physical activity than omnivores [23]. In these studies, the maximum oxygen consumption was measured, being 13% in vegetarian women [23] and 12% in vegetarians [24]. As indicated by several authors in the literature, this variable is clearly marked as a performance marker in performance athletes, so that improvements of approximately 12% in this modality can clearly mark the result in these tests [23,24].

In relation to energy expenditure, it increased by 50 kcal/week and decreased by 80 kcal/week in omnivorous athletes, even though they performed the same exercise protocol [24].

In an incremental cycloergometer test, vegetarian athletes obtained improvements of 3.5 mL/kg/min with respect to VO2máx and a higher VO2máx in a test at submaximal intensities with respect to omnivorous athletes, and, in the submaximal endurance test performed on a cycloergometer at 70% maintaining 70–80 rpm, improvements of 6.1 min were obtained in vegetarian athletes [26]. Possibly, these improvements in the VO2máx variable are related to the improvement of body composition parameters obtained thanks to this type of diet [22,26,30,31].

Improvements in endurance sports in vegetarian athletes may be due to effects on the number of mitochondria, capillary density, and hemoglobin concentration; however, specific studies in which these parameters are measured and evaluated would be necessary to indicate the origin of the improvements [33].

#### 4.3.2. Strength Training

In relation to strength, no significant differences were shown in the quadriceps extension and shoulder press exercises, using the 1 RM technique [26]. As for muscle power, improvements of 21 W in muscle power were obtained in a one-hour test at 60% maximum HR in ovolactovegetarian athletes compared to omnivores [24]. However, there were no significant differences between the groups in average or maximal power in a four-sprint test with a cycloergometer, but maximal power was significantly higher in the first two sprints [28]. Specific research is needed in which tests and/or protocols for the improvement of strength parameters are carried out and can be evaluated.

### 4.4. Metabolism

Another variable that was measured and is related to performance is macronutrient oxidation. Although there were no significant differences in fats or proteins, there were differences in carbohydrates, remaining in vegetarians at 0.3 mL/kg/min and decreasing in omnivores from 0.3 mL/kg/min to 0.1 mL/kg/min [24]. Furthermore, there were no significant differences in the respiratory quotient [24].

However, a review of studies comparing athletes on omnivorous or vegetarian diets found that there were no significant differences between the groups, and that the exercise protocols were very different [34].

#### 4.4.1. Energy Intake

**Figure 8:**
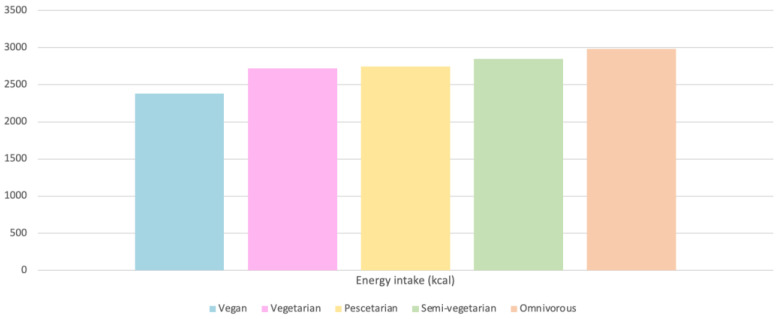
Energy intake as a function of diet [31].

Daily calorie intake varies according to the type of diet of the subjects, with the lowest to highest intakes being those of vegans (2383 kcal), vegetarians (2722 kcal), pesco-vegetarians (2744 kcal), semi-vegetarians (2849 kcal), and, finally, omnivores (2985 kcal) (Figure 8) [31].

In relation to athletes, energy consumption varies depending on the time of the season, being higher during the competitive period corresponding to months 6 and 9 [32].

#### 4.4.2. Macronutrient Intake

**Figure 9:**
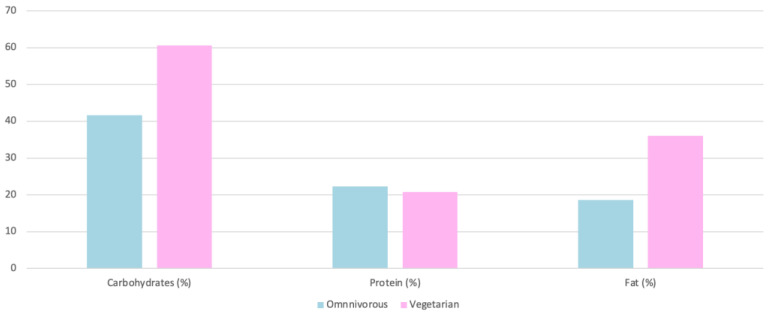
Macronutrient intake as a function of diet [26].

**Figure 10:**
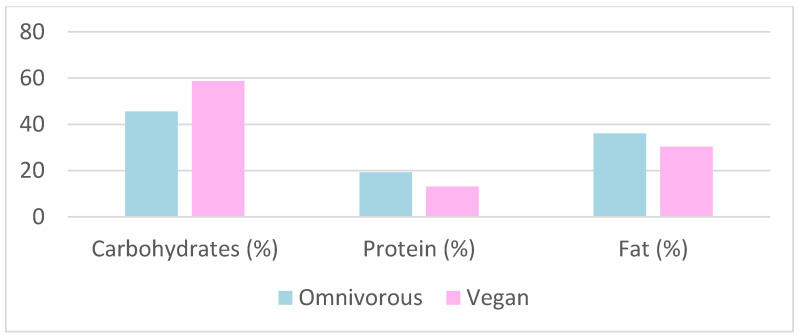
Macronutrient intake as a function of diet [28].

Vegetarians consume a higher amount of carbohydrates (343 g), compared to omnivores, who consume at least 322 g [31]. The same was observed in other studies, with vegetarians consuming 5%, 7.7%, and 17.9% more than omnivores, respectively [23,26,28] (Figure 9 and Figure 10). The energy intake per week is higher in vegetarian women than in omnivorous women (21 kcal/kg/week), and in vegetarian men than in omnivorous men (17 kcal/kg/week) [23].

The Association of Sports Medicine and the College of Dietitian Nutritionists of Canada recommend a carbohydrate intake of 5–10 g/kg/day for athletes, depending on the volume and intensity of training, as well as the competition period.

**Figure 11:**
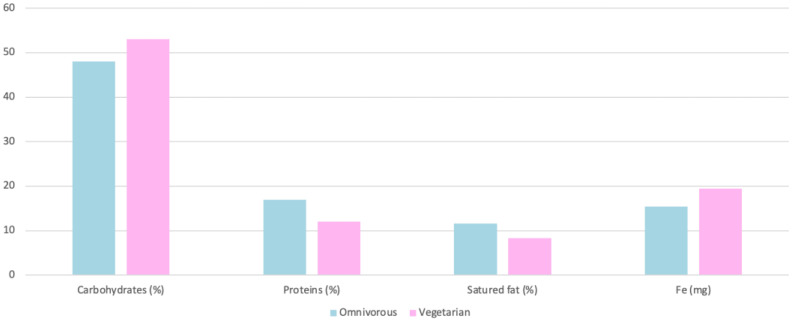
Macronutrient and iron intake by diet [23].

**Figure 12:**
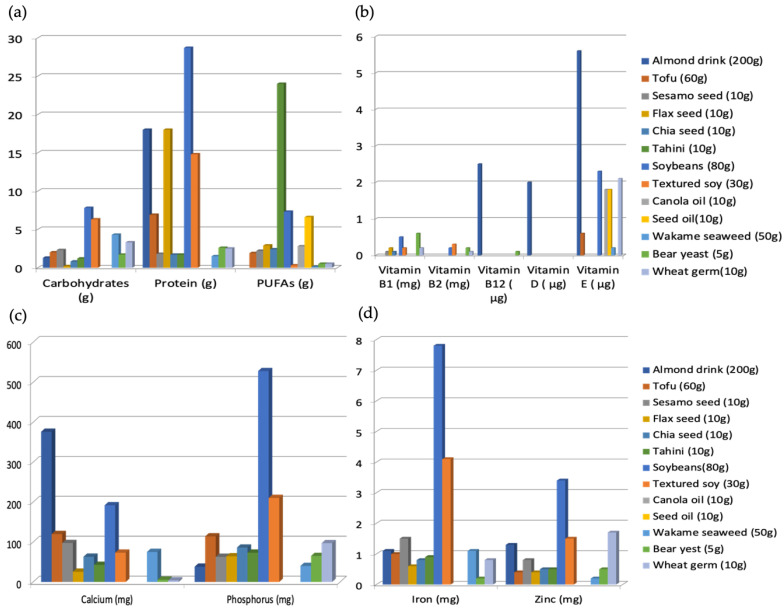
Quantity of macronutrient vitamins and minerals in different foods suitable for vegetarians and vegans [36]: (**a**) macronutrients; (**b**) vitamins; (**c**) calcium and phosphorous; (**d**) iron and zinc.

The intake protein was 93 g in vegetarians and 112 g in omnivores [31]. Protein intake was 12% in vegetarians and 17% in omnivores, with significant differences in both cases. [23] (Figure 11). However, there are many food products suitable for vegetarians that contain high amounts of protein (Figure 12). In relation to protein intake, omnivores consume 9.2% more protein than vegetarians [26]. In relation to endurance athletes, the recommended intake is between 1.2 and 1.7 g/kg/day [35]. Protein intake during the first three months of the season represents 20.37% of the total energy intake, and, in months 6 and 9, it increases to 21.7%, which corresponds to 2.2 g/kg/day [32].

Protein intake is lower in vegetarian athletes, but there are many foods suitable for athletes who follow this type of diet, for example, textured soybeans contain 28.7 g/80 g, almond drink 18 g/200 g, or flax seeds 18 g/10 g, among many others [36].

The consumption of saturated fats represents 8.3% in vegetarians and 11.6% in omnivores [23] (Figure 11). Total fats were assessed and accounted for 31% in vegetarians and 36% in omnivores [31]. Furthermore, total and saturated fat consumption is higher in omnivores than in vegetarians, 7.1% and 136.5%, respectively [26,28] (Figure 9 and Figure 10). The amount required for endurance athletes is 2 g/kg/day or 1.6 g/kg/day [32,35]. The average intake of essential fatty acids in endurance athletes is increased by 4% during the third and sixth months of the season [32].

Although it is widely believed that a vegetarian athlete hardly consumes any fat there are many vegetarian foods with a high fat content such as tahini, 24 g/10 g, soybeans 7.3 g/80 g, and olive oil 90 g/100 mL [36].

#### 4.4.3. Micronutrient Intake

We will consider the following minerals: iron (Fe) and calcium (Ca), which have been analyzed in the different studies. For endurance athletes, iron consumption is higher in vegetarians (19.4 mg) than in omnivores (15.4 mg), which represents a significant difference [23] (Figure 11). Whereas red meat is generally thought to be the most iron-containing food, there are iron-rich foods that are suitable for vegetarians (Figure 12). Some of the foods that can be consumed by vegetarian athletes and that contain a significant amount of iron are texturized soybeans, 4.1 mg/30 g, soybeans 7.8 mg/80 g, or wakame seaweed 1.1 mg/50 g [36].

Calcium consumption is higher in vegetarians than in omnivores, with a significant difference of 266 mg per day [31]. Vitamin B12 intake is also noteworthy, with omnivores consuming 2.86 mcg more than vegetarians [26]. Although we normally think of dairy products when we talk about calcium, it is true that there are other beverages or foods that have an equal or greater amount, as is the case with almond drink, 376 mg/200 g, or tofu with 120 mg/60 g [36].

We analyzed cyanocobalamin (vitamin B12), which is the most frequently deficient in vegetarian athletes. This vitamin is not generally present in foods of vegetable origin; however, athletes can obtain it through meat analogs or algae and enriched foods, covering up to 100% of the requirements [36].

A well-planned vegetarian diet with the right combination of foods, which satisfies nutritional requirements, can be optimal for athletes to achieve high performance [37,38,39,40].

An interesting future line of research would be to determine whether the vegetarian diet is optimal for improving performance in strength sports and to evaluate the diets of vegetarian athletes to determine whether they achieve the dietary reference intakes (RDI) of different micronutrients.

## 5. Conclusions

In relation to performance, athletes on a vegetarian diet obtained significantly higher values of relative oxygen consumption and maximum power, compared to omnivores. However, no significant differences were found in strength-related parameters.

Physical fitness was higher in vegetarian women, although no significant differences were shown. Finally, and in relation to dietary intake, vegetarian or vegan athletes consumed significantly more carbohydrates and less protein and saturated fat. Therefore, it is important for athletes to plan a diet that meets their nutritional needs according to the type of sport, as well as the period of the season they are in.

## Figures and Tables

**Table 1:** Types of diets.

	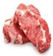	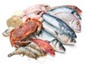	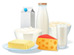	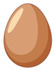	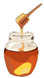	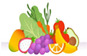
**Non-vegetarian**						
**Omnivorous**	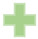	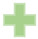	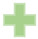	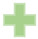	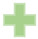	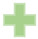
**Vegetarian-based**						
**Ovo-lacto-vegatarian**	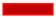	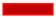	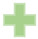	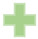	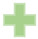	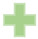
**Ovo-vegetarian**	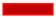	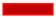	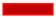	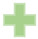	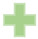	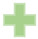
**Lacto-vegetarian**	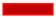	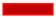	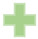	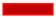	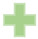	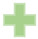
**Vegan**	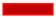	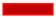	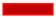	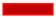	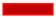	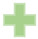

**Table 2:** List of variables of the results.

Physical health	Quality of life questionnaire (WHOQOL-BREF). Psychological well-being and social relationships. % of subjects performing one endurance modality or the other according to sex and diet.
Body composition	Body mass. Lean mass. Fat mass.
Performance	Relative, absolute, and maximum oxygen consumption. Maximum number of extensions and push-ups. Maximum performance. Muscular strength. Respiratory Coefficient. Macronutrient oxidation. International Physical Activity Questionnaire. Fatigue index. Average and peak power.
Energy, macronutrient, and micronutrient intake	Basal metabolic rate (BMR). Dietary profile. Dietary intake. Energy, macronutrients, and Fe intake.

**Table 3:** Table of results for each study.

Ref.	Subjects	Program	Variables	Performance/Health			
[25]	281 subjects 123 omnivorous diet 158 vegetarian diet Control group: 10 km 2 diet groups: Omnivores and vegetarians * or vegans 3 distance groups: 10 km Half marathon Marathon/Ultra marathon	WHOQOL-BREF Quality of Life Study	Physical health	Physical health: 84.6% omnivorous women 85.4 vegetarian women 87.4 male omnivores 87% male vegetarians 10 km fem: 84.3% 10 km men: 87.4% ½ Marathon fem: 87.8% ½ Marathon men: 86.5% Women’s marathon: 86.5% Men’s marathon: 86.4%			
[23]	NCAA Division 1 Athletes 27 vegetarians * 43 omnivores	Their meals for the last 7 days were recorded. Oxygen consumption → Bruce protocol Fatigue → Borg RPE Leg extensions and push-ups → dynamometer	Body composition	Body mass Vegetarian *: −11% Lean mass Vegetarian *: −7% Fat mass Vegetarian: 25.5% Omnivores: 26.9% Vegetarians: 19.2% Omnivores: 19.2%			
			Relative/absolute maximal oxygen uptake	Relative maximal oxygen uptake Vegetarian *: +13% Absolute Maximum Oxygen Consumption No significant differences			
			Maximum number of extensions and push-ups.	Maximum number of extensions and push-ups No significant differences			
			Energy, macronutrients, and Fe intake	Energy consumption per week Vegetarian: 106.1 kcal/kg/week Omnivores: 85.6 kcal/kg/week Vegetarians: 108.8 kcal/kg/week Omnivores: 91.7 kcal/kg/week Carbohydrates * Vegetarians: 53% Omnivores: 48% Protein *: Vegetarians: 12% Omnivores: 17% Saturated fats: Vegetarians: 8.3% Omnivores: 11.6% Iron (Fe) * Vegetarians: 19.4 mg Omnivores: 15.4 mg			
[24]	74 subjects with type 2 diabetes mellitus Treatment → oral hypoglycemic agents. 2 groups: Lactovegetarians (maximum 1 daily serving of skimmed dairy products) Control (omnivorous diet)	Duration: 12 weeks Diet: Vegetarian: 60% HC, 15% protein, 25% fat Omnivore: 50% HC, 20% protein, 30% fat Exercise: Frequency: 2 days/week Intensity: 60% HR max Duration: 1 h	Maximum performance Maximum oxygen consumption Respiratory Coefficient Fasting fat and protein carbohydrate oxidation Adherence to exercise	Performance: Vegetarians: +23 W Control: +4 W Maximum oxygen consumption Vegetarians: 3 mL/kg/min Control: −1 mL/kg/mi Respiratory quotient Did not change significantly in any of the groups Oxidation of fats and proteins No significant change in either group Fasting carbohydrate oxidation Vegetarians: 0.3–0.3 mL/kg/min Control: 0.4–0.1 mL/kg/min Adherence to exercise Vegetarians: 90.3%. Control Group: 80.6%			
			BMR	BMR Vegetarians: 2050–2100 kcal Control: 1950–1970 kcal			
[27]	Subjects: 2864 Omnivores (OMV): 1272 Vegetarians * (VEGT): 598 Vegans (VEG): 994	Standardized questionnaire of the NURMI study	% of subjects performing one endurance modality or the other according to sex and diet	Women			
					MVNO	VEGT	VEG
				<21 km	23%	29%	30%
				MM	40%	38%	43%
				M/UM	37%	33%	27%
				Men			
					MVNO	VEGT	VEG
				<21 km	11%	14%	14%
				MM	29%	32%	31%
				M/UM	60%	54%	55%
				MM: half marathon; M: marathon; UM: ultra marathon			
[26]	56 healthy young women Vegan: 28 Omnivores: 28	Cycloergometer endurance test: Warm-up → 2 min at 50 W Every 2 min →↑ 25 W Frequency → 70–80 rpm Test of strength: 1 RM test on leg and chest presses	VO_2_ max. Muscular strength Dietary profile	VO_2_ max: ↑ 3.5 mL/kg/min in vegans than in omnivores. Submaximal endurance: ↑ 6.1 min in vegans than in omnivores. No significant differences in strength. Dietary profile: Carbohydrates: ↑ 7.7% in vegans Protein: ↑ 9.2% in omnivores Total fat: no significant differences Saturated fats: ↑ 7.1% in omnivores Vitamin B12: ↑ 2.86 mcg in omnivores Iron: ↑ 13.3 mg in omnivores			
[28]	18 subjects: 9 vegans 9 omnivores	Familiarization session Fasting 10–12 h Warm up: 5 min at 80 rpm + 4 s sprints at the end of minutes 2 and 4. 3 min break Main part: 4 sprints of 30 s 5 min rest between sprints	International Physical Activity Questionnaire Fatigue index Average and peak power	No significant differences in any parameter of physical activity volume/intensity. There was no significant difference in fatigue. Peak power: ↑ in the 1st and 2nd sets compared to the 3rd and 4th sets. No significant differences between groups. Average power: No significant differences between series or between groups.			
			Dietary intake	Dietary intake: Carbohydrates: ↑ 17.9% in vegans Protein: no significant differences Fat: ↑ 21.4% in omnivores Saturated fats: ↑ 36.5% in omnivores			

* Vegetarians without specifying whether it is a lactovegetarian or ovolactovegetarian diet.

**Table 4:** Methodological assessment PEDro scale.

Type of Study		PEDro												Conflict of Interests
		1	2	3	4	5	6	7	8	9	10	11	TOTAL	NO
[25]	Cross-sectional-study	+	-	+	+	-	-	-	+	+	+	+	7/10	NO
[23]	Cross-sectional study	+	-	+	+	-	-	-	-	+	+	+	6/10	NO
[24]	Parallel Randomized Trial	+	+	-	+	-	-	-	+	+	+	+	7/10	N/A
[27]	Cross-sectional-study	+	-	-	+	-	-	-	-	+	+	+	5/10	NO
[26]	Control-trial	+	-	-	+	-	-	-	+	+	+	+	6/10	NO
[28]	Control-trial	+	-	-	+	-	-	-	+	+	+	+	6/10	N/A

1: Eligibility criteria were specified; 2: subjects were randomly allocated to groups; 3: allocation was concealed; 4: the groups were similar at baseline regarding the most important prognostic indicators; 5: blinding of all subjects; 6: blinding of all therapists who administered the therapy; 7: blinding of all assessors who measured at least one key outcome; 8: >85% outcomes of the subjects initially allocated to groups; 9: data for at least one key outcome by “intention to treat”; 10: between-group statistical comparisons; 11: point measures and measures of variability; N/A: not available; + sign means that it meets the quality criteria; - sign means that it does not meet the quality criteria.

## Supplementary Materials

The following supporting information can be downloaded at: https://www.mdpi.com/article/10.3390/nu15214703/s1, Suplementary Material S1: PRISMA Checklist [41].
